# Childhood Wheezing, Asthma, Allergy, Atopy, and Lung Function: Different Socioeconomic Patterns for Different Phenotypes

**DOI:** 10.1093/aje/kwv045

**Published:** 2015-10-06

**Authors:** Bruna Galobardes, Raquel Granell, Jonathan Sterne, Rachael Hughes, Cilia Mejia-Lancheros, George Davey Smith, John Henderson

**Keywords:** asthma, atopy, childhood, inequalities, phenotypes, socioeconomic position

## Abstract

Identifying preventable exposures that lead to asthma and associated allergies has proved challenging, partly because of the difficulty in differentiating phenotypes that define homogeneous disease groups. Understanding the socioeconomic patterns of disease phenotypes can help distinguish which exposures are preventable. In the present study, we identified disease phenotypes that are susceptible to socioeconomic variation, and we determined which life-course exposures were associated with these inequalities in a contemporary birth cohort. Participants included children from the Avon Longitudinal Study of Parents and Children, a population-based birth cohort in England, who were born in 1991 and 1992 and attended the clinic at 7–8 years of age (*n* = 6,378). Disease phenotypes included asthma, atopy, wheezing, altered lung function, and bronchial reactivity phenotypes. Combining atopy with a diagnosis of asthma from a doctor captured the greatest socioeconomic variation, including opposing patterns between phenotype groups: Children with a low socioeconomic position (SEP) had more asthma alone (adjusted multinomial odds ratio = 1.50, 95% confidence interval: 1.21, 1.87) but less atopy alone (adjusted multinomial odds ratio = 0.80, 95% confidence interval: 0.66, 0.98) than did children with high SEP. Adjustment for maternal exposure to tobacco smoke during pregnancy and childhood exposure to tobacco smoke reduced the odds of asthma alone in children with a low SEP. Current inequalities among children who have asthma but not atopy can be prevented by eliminating exposure to tobacco smoke. Other disease phenotypes were not socially patterned or had SEP patterns that were not related to smoke exposure.

A large number of life-course factors and exposures have been associated with asthma (1–4). Determining which are causal and amenable to prevention has proved difficult, and asthma continues to be poorly understood, partly because of the difficulty in defining asthma phenotypes that identify homogeneous disease groups (5, 6). Both classical and more recently reported phenotypes are defined by clinical and biological characteristics of this condition (7), such as the presence of atopy (8), altered lung function (8, 9), and variability of wheezing symptoms over time (10, 11), or include the genetic variability of different phenotype groups (12).

A socioeconomic patterning of asthma and allergies has been identified in a number of studies (13–18), in which great variability in the magnitude and direction of these inequalities was reported (16, 18, 19). Given this variability, demonstrating the existence and direction of socioeconomic inequalities in the prevalence of asthma and atopy in a contemporary birth cohort is important. Furthermore, ascertaining the socioeconomic patterning of a comprehensive list of disease phenotypes could help identify biological and clinical characteristics that are susceptible to socioeconomic variation and thus susceptible to intervention.

Understanding the exposures that mediate these socioeconomic position (SEP) patterns could help identify avoidable exposures. These exposures are likely to be relevant to the overall burden of disease as well as to the inequality between groups. This has been demonstrated in cardiovascular research in which it has been shown that the traditional cardiovascular risk factors that account for most of the disease burden in the population also underpin the inequality in cardiovascular health between socioeconomic groups (20, 21). The aims of our study were to describe the socioeconomic patterning of asthma and atopy in an extensive list of different phenotype definitions and to establish life-course characteristics and exposures associated with these socioeconomic patterns.

## METHODS

### Study participants

The Avon Longitudinal Study of Parents and Children (ALSPAC) is a prospective, population-based birth cohort study in England that aims to examine the genetic and environmental determinants of health (22, 23). A total of 14,541 pregnant women residing in 3 Bristol-based health districts who had expected dates of delivery between April 1, 1991, and December 31, 1992, were recruited. Our eligible study sample included singleton children (*n* = 14,273) with a gestational age of 37 weeks or more (*n* = 13,024) for whom we had information about paternal educational levels (*n* = 10,009) and who attended the Avon Longitudinal Study of Parents and Children clinic at 7–8 years of age (*n* = 6,378).

Ethics approval for the study was obtained from the Avon Longitudinal Study of Parents and Children Law and Ethics Committee and the United Kingdom National Health Service local research ethics committee. Written informed consent was obtained from the parents or main caregivers for all measurements.

### Data collection and variable definitions

#### Asthma and allergy phenotypes

When a child was seen at 7–8 years of age (mean age, 7.5 years), participating parents were asked whether their child had suffered asthma, eczema, or hay fever in the past 12 months to characterize phenotypes based on recent self-reported symptoms. They were also asked whether their child had ever been given a diagnosis of asthma by a doctor to classify children by asthma status. At 7–8 years of age, child participants underwent skin-prick testing for a core panel of 6 common allergens (ALK-Abello A/S, Hørsholm, Denmark). Atopy was classified as at least 1 positive response (mean weal size ≥2 mm) to mixed grass, house dust mite, or cat allergens with no response to negative control solution and a response to positive control (histamine).

Combined asthma and atopy phenotypes were based on diagnoses of asthma by a doctor and results of skin-prick tests. The phenotypes identified were children with no asthma and no atopy (reference group), children with asthma alone, children with asthma and atopy, and children with atopy alone.

Wheezing phenotypes were based on the temporal pattern of wheezing in children reported by the parent at 0.5, 1.5, 2.5, 3.5, 4.5, 5, and 6.75 years after birth using latent class analysis (11). The phenotypes identified were never or infrequent wheeze (reference group), transient early wheeze, prolonged early wheeze, intermediate-onset wheeze, late-onset wheeze, and persistent wheeze.

#### Lung function and bronchial responsiveness

Lung function was measured using a hand-held spirometer (Vitalograph 2120; Vitalograph Ltd., Maids Moreton, United Kingdom) when the children were 8–9 years of age, following American Thoracic Society criteria (24). Flow-volume curves were reviewed by a respiratory physician. Forced expiratory volume in 1 second (FEV_1_), forced vital capacity, and midbreath forced expiratory flow were converted to sex-, age-, and height-adjusted standard deviation units (25). Bronchial responsiveness was measured with a test of bronchial reactivity to methacholine using the method of Yan et al. (26) and expressed as the dose-response slope of FEV_1_ (percentage decline from baseline) per micromole of methacholine for each subject. Children were classified as having a positive bronchial responsiveness test if their measurements fell within the upper tertile of the distribution of those with a measurable cumulative dose of histamine that causes a 20% fall in FEV_1_ (*n* = 5,428).

#### Socioeconomic position

We investigated a long list of indices of SEP: maternal and paternal educational levels, maternal and paternal occupational class (based on the Registrar General's Social Class Classification), household income, housing tenure, and a composite indicator combining all previous indicators with principal component analysis. Each indicator measures different but related aspects of socioeconomic stratification (27, 28). We did not have an a priori best indicator, and we aimed to report differences between indicators if these existed. However, there were no important differences in the SEP pattern identified by each indicator; the conclusions remained the same regardless of the indicator used. Therefore, we present results using paternal educational level obtained through maternal report at 32 weeks' gestation because there were fewer missing values in this indicator and it captured the largest inequalities in all phenotypes. Paternal educational level was categorized as high (university degree or higher), medium (A levels; education up to 18 years), or low (O levels or below; educational level attained at 16 years of age, including vocational education).

#### Life-course exposures and other child and parental characteristics

A long list of environmental exposures and explanatory characteristics were investigated based on current knowledge and availability (Web Appendix 1, available at http://aje.oxfordjournals.org/). We also investigated grouped exposures of life-course exposure to tobacco smoke and life-course exposure to hygiene hypothesis-related variables.

### Statistical analysis

The eligible study sample included participants for whom we had available information about paternal educational level and who attended the clinic at age 7–8 years, when skin-prick reaction and lung function were measured (*n* = 6,378). There were varying degrees of missing values because of the long-term follow-up. In order to increase efficiency and minimize selection bias, we used multivariable multiple imputation to impute missing variables for eligible participants (detailed information is available in Web Appendix 1 and Web Table 1).

Multivariable linear, logistic, and multinomial regression models were used to investigate associations of linear (lung function), binary (asthma, eczema, or hay fever in the past 12 months; doctor diagnosis of asthma; atopy; and bronchial hyper-responsiveness), and categorical (with more than 2 groups: combined asthma and atopy phenotypes and wheezing phenotypes) outcomes with paternal educational level. There were no differences in the socioeconomic patterning of outcomes by sex, and all results are grouped adjusting for sex and age. Associations are presented with the regression coefficient for continuous outcomes (linear regression), odds ratio for binary outcomes, and multinomial odds ratio (MOR) for categorical outcomes. The MOR was calculated for each category of the outcome variable compared with the reference category.

The analytical strategy was to report the socioeconomic patterning of all different phenotypes, to determine the SEP pattern of explanatory factors and exposures, and to calculate the attenuation of the SEP-phenotype pattern found after accounting for each exposure or group of exposures. Only exposures that attenuated the SEP-phenotype pattern by 5% or more were considered. We calculated the attenuation in the association between paternal educational level and disease phenotype due to 1 or several factors or exposures as the percentage change in the MOR (or odds ratio), as

$$((\text{MO}{\text{R}}_{1}-\text{MO}{\text{R}}_{1+\mathrm{one}/\mathrm{several}\;\;\mathrm{factors}})/(\text{MO}{\text{R}}_{1}-\;1)\;\times \;100),$$

where “1” refers to a model including paternal educational level, age, and sex. All analyses were carried out with Stata software, version 13.0 (StataCorp LP, College Station, Texas).

## RESULTS

A total of 6,378 participants had information available about paternal educational level and attended the clinic at 7–8 years of age. Table 1 shows the distribution of outcome and exposure variables. Approximately half the participants were boys, and 23.8% of children had fathers with a university degree. Of these, 11.1% had asthma, 17.7% had eczema, and 9.2% had hay fever in the previous 12 months as reported by their parents. Approximately 20% of the parents reported that their children had ever received a doctor's diagnosis of asthma, and a similar proportion of children were atopic according to a skin-prick test. Similar proportions of children were classified as having asthma alone and atopy alone (11.9% and 13.0%, respectively), and 7.6% had both. Bronchial hyperresponsiveness was found in 16.7% of the study participants. The distributions of outcome and exposure variables in the imputed data set were very similar to those in the original data set (Web Table 1); therefore, imputed data are used hereafter.

**Table 1.** Characteristics of Children and Their Parents in the Original Data Set^a^, Avon Longitudinal Study of Parents and Children, 1991–1999

Characteristic or Risk Factor	No. in Data Set	No. With Characteristic	%	Mean (SD)
Male sex		3,201	50.2	
Child's age, years	6,378			7.5 (0.18)
Paternal educational level^b^	6,378			
High		1,520	23.8	
Medium		1,901	29.8	
Low		2,957	46.4	
	*Outcomes*			
Asthma in the past 12 months	5,563	616	11.1	
Eczema in the past 12 months	5,561	982	17.7	
Hay fever in the past 12 months	5,541	510	9.2	
Doctor diagnosis of asthma	5,530	1,082	19.6	
Atopy (positive skin-prick test)	5,402	1,105	20.5	
Lung function, *z* score				
FVC	5,089			0.02 (1.0)
FEV_1_	5,015			0.03 (1.0)
FEF_25-75_	5,089			0.03 (1.0)
Bronchial hyperresponsiveness	5,428	906	16.7	
Combined asthma-atopy phenotype^c^	4,700			
No asthma and no atopy		3,176	67.6	
Asthma alone		559	11.9	
Atopy alone		609	13.0	
Asthma and atopy		356	7.6	
Wheezing phenotype^d^	6,253			
Infrequent wheeze			63.0	
Transient early wheeze			12.4	
Prolonged early wheeze			9.2	
Intermediate-onset wheeze			2.3	
Late-onset wheeze			6.1	
Persistent wheeze			6.9	
	*Exposures/Factors*			
Maternal asthma	6,200	705	11.4	
Paternal asthma	4,757	614	12.9	
Maternal eczema or hay fever	6,116	2,808	45.9	
Paternal eczema or hay fever	4,663	1,863	40.0	
During pregnancy				
Maternal smoking	6,091			
None		5,069	83.2	
1–2 trimesters		396	6.5	
Throughout 3 trimesters		626	10.3	
Paternal smoking for 1–2 trimesters	5,978	1,825	30.5	
Environmental tobacco smoke >1 hour per day	5,307	2,348	44.2	
Crowding^e^	6,216			
<0.5		1,928	31.0	
0.50–0.75		2,547	41.0	
0.76–0.99		979	15.7	
≥1		762	12.3	
Pet ownership	6,277	3,620	57.7	
Pest exposure at home	6,277	899	14.3	
Gas cooking	6,206	3,348	53.9	
Bleach or dye use daily or most days	6,300	1,049	16.7	
Cold or very cold bedroom	6,177	964	15.6	
Fairly or very serious mold in the home	6,202	114	1.8	
At birth				
Maternal age	6,378			29.3 (4.4)
Paternal age	4,512			31.6 (5.5)
Gestational age, weeks				39.8 (1.3)
Birth weight, *z* score	6,307			0.07 (0.98)
Childhood				
Other children in the household at birth	6,080	2,726	44.8	
Breastfeeding duration	5,989			
>3 months		3,125	52.2	
1–3 months		925	15.4	
<1 month		1,939	32.4	
Maternal smoking	5,588	907	16.2	
Paternal smoking	5,110	1,081	21.1	
Environmental tobacco smoke >1 hour per day	5,500	754	13.7	
Cotinine level, ng/mL	4,316			1.2 (1.2)
Day care attendance at age 15 months	5,991			
No		4,674	78.0	
Other caregiver in the home		896	15.0	
Nursery		421	7.0	
Crowding at age 21 months^e^	5,644			
<0.5		549	9.7	
0.50–0.75		2,591	45.9	
0.76–0.99		1,417	25.1	
≥1		1,087	19.3	
Pet ownership	5,537	3,975	71.8	
Pest exposure at home	6,087	933	15.3	
Gas cooking	5,642	3,495	61.9	
History of chest infections	5,599	578	10.3	
Cold or very cold bedroom	5,609	298	5.3	
Fairly or very serious mold in the home	2,855	79	2.8	

Abbreviations: FEF_25-75_, midbreath forced expiratory flow; FEV_1_, forced expiratory volume in 1 second; FVC, forced vital capacity; SD, standard deviation.

^a^ Distribution of imputed and original data set in Web Table 1.

^b^ High indicates a university degree; medium indicates A levels (education up to 18 years); and low indicates O levels or below (education attained at 16 years of age, including vocational education).

^c^ Combined phenotype using a maternal report of a diagnosis of asthma by a doctor and atopy results obtained through skin-prick testing.

^d^ Weighted frequency obtained using latent class analysis.

^e^ A crowding index was calculated by dividing the number of people in the household by the number of rooms, both of which were reported by questionnaire. See Web Appendix 1 for more details.

### Socioeconomic patterning of different disease phenotypes

Children with a lower SEP were more likely to have had asthma in the past 12 months or to have been diagnosed with asthma by a doctor, but they were less likely to have had eczema in the past 12 months and were less likely to be atopic (Table 2). Hay fever in the past 12 months and bronchial hyperresponsiveness were not socioeconomically patterned (Table 2). Lung function was not socioeconomically patterned, with the exception of a slightly lower FEV_1_ in children with a low SEP (Table 3).

**Table 2.** Association of Paternal Educational Level With Different Asthma, Allergy, and Respiratory Outcomes (*n* = 6,378^a^), Avon Longitudinal Study of Parents and Children, 1991–1999

Paternal Educational Level^b^	Exhibited Symptoms in the Past 12 Months						Diagnosis of Asthma by Doctor		Atopy (Positive Skin-Prick Test)		Bronchial Hyperresponsiveness	
	Asthma		Eczema		Hay Fever							
	aOR^c^	95% CI	aOR^c^	95% CI	aOR^c^	95% CI	aOR^c^	95% CI	aOR^c^	95% CI	aOR^c^	95% CI
High	1.00	Referent	1.00	Referent	1.00	Referent	1.00	Referent	1.00	Referent	1.00	Referent
Medium	1.07	0.85, 1.35	0.82	0.69, 0.99	1.11	0.87, 1.42	1.14	0.95, 1.37	1.00	0.84, 1.20	0.96	0.79, 1.17
Low	1.32	1.07, 1.63	0.80	0.68, 0.95	1.02	0.81, 1.28	1.36	1.15, 1.60	0.84	0.71, 0.98	0.98	0.82, 1.17
Linear trend	1.16	1.04, 1.29	0.90	0.83, 0.98	1.00	0.89, 1.12	1.17	1.08, 1.27	0.91	0.84, 0.98	0.99	0.91, 1.08

Abbreviations: aOR, adjusted odds ratio; CI, confidence interval.

^a^ Generated from multiple imputation with 25 imputed data sets.

^b^ High indicates a university degree; medium indicates A levels (education up to 18 years); and low indicates O levels or below (education attained at 16 years of age, including vocational education).

^c^ Adjusted for age and sex.

**Table 3.** Mean Differences in Lung Function Across Paternal Educational Level (*n* = 6,378^a^), Avon Longitudinal Study of Parents and Children, 1991–1999

Paternal Educational Level^b^	Measure of Lung Function					
	FVC		FEV_1_		FEF_25-75_	
	Mean Difference^c^	95% CI	Mean Difference^c^	95% CI	Mean Difference^c^	95% CI
High	0.00	Referent	0.00	Referent	0.00	Referent
Medium	−0.01	−0.08, 0.07	−0.02	−0.10, 0.05	−0.02	−0.10, 0.05
Low	−0.06	−0.12, 0.01	−0.07	−0.14, −0.002	−0.04	−0.11, 0.02
Linear trend	−0.03	−0.06, 0.004	−0.04	−0.07, −0.003	−0.02	−0.06, 0.01

Abbreviations: CI, confidence interval; FEF_25-75_, midbreath forced expiratory flow; FEV_1_, forced expiratory volume in 1 second; FVC, forced vital capacity.

^a^ Generated from multiple imputation with 25 imputed data sets.

^b^ High indicates a university degree; medium indicates A levels (education up to 18 years); and low indicates O levels or below (education attained at 16 years of age, including vocational education).

^c^ Adjusted for age and sex.

Opposing socioeconomic patterns were found within categories of the combined asthma and atopy phenotypes (Table 4): Children with a low SEP were more likely to have asthma alone but less likely to have atopy alone. There was no socioeconomic patterning among those who had both asthma and atopy. With regard to wheezing patterns, children with low SEP were more likely to have persistent wheeze (Table 5).

**Table 4.** Associations of Paternal Educational Level With the Combined Asthma and Atopy Phenotype (*n* = 6,378^a^), Avon Longitudinal Study of Parents and Children, 1991–1999

Paternal Education Level^b^	Asthma Alone		Asthma and Atopy		Atopy Alone		*P* for Heterogeneity^c^
	aMOR^d^	95% CI	aMOR^d^	95% CI	aMOR^d^	95% CI	
High	1.00	Referent	1.00	Referent	1.00	Referent	
Medium	1.15	0.90, 1.47	1.11	0.84, 1.47	0.97	0.78, 1.21	0.51
Low	1.50	1.21, 1.87	1.06	0.82, 1.36	0.80	0.66, 0.98	<0.00001
Linear trend	1.24	1.11, 1.38	1.02	0.90, 1.15	0.89	0.81, 0.98	<0.00001

Abbreviations: aMOR, adjusted multinomial odds ratio; CI, confidence interval.

^a^ Generated from multiple imputation with 25 imputed data sets.

^b^ High indicates a university degree; medium indicates A levels (education up to 18 years); and low indicates O levels or below (education attained at 16 years of age, including vocational education).

^c^ Test for equality of coefficients across outcome groups.

^d^ Compared with the group that had no asthma and no atopy. The models were adjusted for age and sex.

**Table 5.** Association of Paternal Educational Level With Wheezing Phenotypes^a^ (*n* = 6,253), Avon Longitudinal Study of Parents and Children, 1991–1999

Paternal Education Level^b^	Transient Early		Prolonged Early		Intermediate Onset		Late Onset		Persistent		*P* for Heterogeneity^d^
	aMOR^c^	95% CI	aMOR^c^	95% CI	aMOR^c^	95% CI	aMOR^c^	95% CI	aMOR^c^	95% CI	
High	1.00	Referent	1.00	Referent	1.00	Referent	1.00	Referent	1.00	Referent	
Medium	0.97	0.79, 1.20	1.05	0.83, 1.33	0.96	0.61, 1.50	1.12	0.84, 1.48	1.41	1.05, 1.88	0.28
Low	1.00	0.82, 1.21	1.00	0.80, 1.25	0.93	0.62, 1.41	0.93	0.71, 1.22	1.46	1.12, 1.92	0.09
Linear trend	1.00	0.91, 1.10	1.00	0.89, 1.11	0.97	0.79, 1.18	0.95	0.84, 1.08	1.18	1.04, 1.34	0.11

Abbreviations: aMOR, adjusted multinomial odds ratio; CI, confidence interval.

^a^ Weighted frequency obtained using latent class analysis.

^b^ High indicates a university degree; medium indicates A levels (education up to 18 years); and low indicates O levels or below (education attained at 16 years of age, including vocational education).

^c^ Compared with the group that had no or infrequent wheeze and using children's phenotype probabilities as weights. The models were adjusted for age and sex.

^d^ Test for equality of coefficients across outcome groups.

### Exposures and factors attenuating the socioeconomic patterning

#### Combined asthma-atopy phenotype

**Figure 1.**
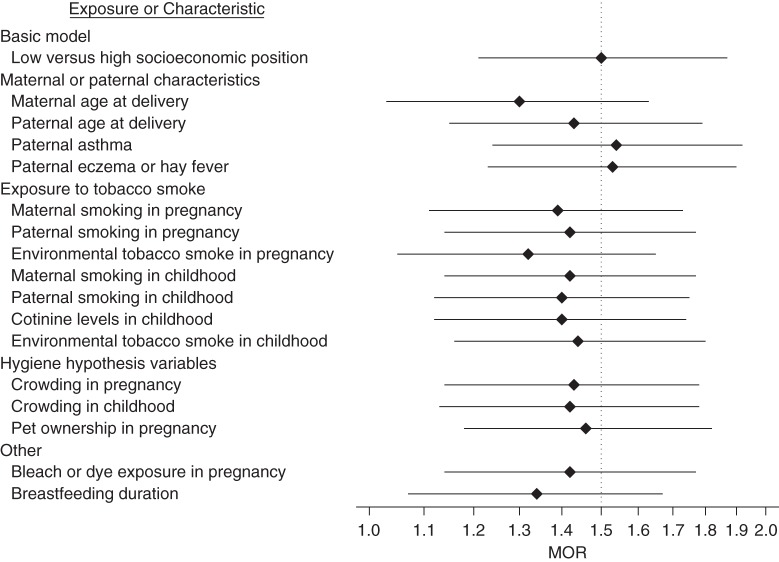
Age- and sex-adjusted multinomial odds ratios (MORs) and 95% confidence intervals for low versus high paternal education in the asthma alone group of the combined asthma and atopy phenotype, before and after additionally adjusting for 1 exposure or characteristic at a time, Avon Longitudinal Study of Parents and Children, 1991–1999. Only exposures that resulted in a change in MOR of 5% or more are shown (*n* = 6,378, generated from multiple imputation with 25 imputed data sets).

**Figure 2.**
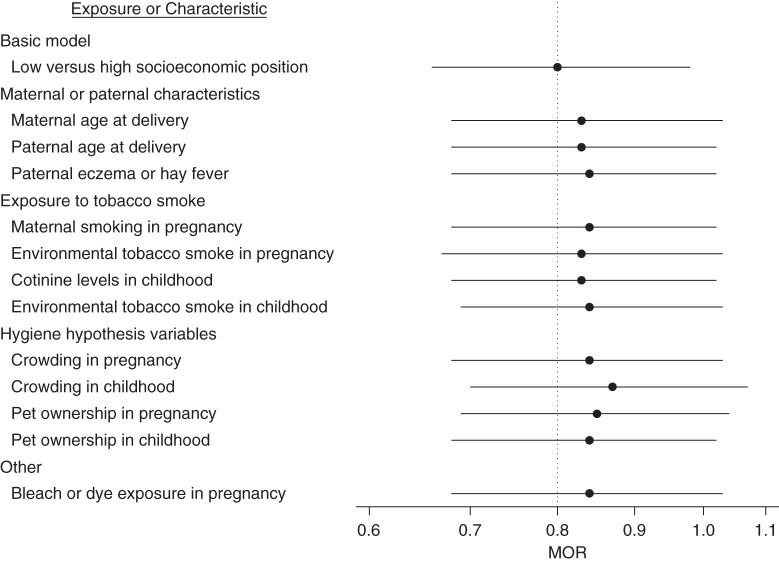
Age- and sex-adjusted multinomial odds ratios (MORs) and 95% confidence intervals for low versus high paternal education in the atopy alone group of the combined asthma and atopy phenotype, before and after additionally adjusting for 1 exposure or characteristic at a time, Avon Longitudinal Study of Parents and Children, 1991–1999. Only exposures that resulted in a change in MOR of 5% or more are shown (*n* = 6,378, generated from multiple imputation with 25 imputed data sets).

Most exposures and factors were socioeconomically patterned (results not shown), were associated with the outcome (in this case the combined asthma-atopy phenotype; Web Table 2), and therefore had the potential to attenuate the SEP pattern observed in this phenotype. Figures 1 and 2 present the age- and sex-adjusted association of low SEP (high was the reference group) and the attenuation of this association by adjustment for each characteristic or exposure. For clarity, only exposures that resulted in an attenuation of 5% or more are shown; the combined asthma-atopy group is not shown because that outcome was not socioeconomically patterned. For asthma alone (Figure 1), no single variable fully attenuated the socioeconomic patterning, and most attenuations were of relatively small magnitude. Higher maternal age at delivery, all smoking-related variables, shorter breastfeeding duration, a higher crowding index, and use of bleach or dye daily or on most days during pregnancy were associated with the highest attenuations (see Web Appendix 1 for detailed information on variables). For atopy alone (Figure 2), point estimates were very similar to the unadjusted estimates after accounting for single characteristics or exposures. There was some tendency for a greater attenuation with variables related to the hygiene hypothesis.

Table 6 shows the attenuation of the SEP pattern after inclusion of grouped life-course exposures to tobacco smoke and hygiene-related variables in the model. Life-course exposure to tobacco smoke was associated with a 44% attenuation of the higher odds of asthma alone associated with low SEP. Most of this attenuation was due to maternal smoking or maternal environmental tobacco smoke exposure during pregnancy. Adding maternal age at delivery or breastfeeding to models with life-course smoking exposure attenuated the association to the null in participants with this phenotype. Exposure to tobacco smoke also attenuated the lower odds of atopy alone associated with low SEP (25%). Hygiene hypothesis exposures attenuated by 60% the lower odds of atopy alone, with no remaining association, and by 28% the higher odds of asthma alone (mostly because of the crowding variables). Adding maternal age at delivery or breastfeeding fully reduced the lower odds of atopy alone in children with a low SEP.

**Table 6.** Low Versus High Paternal Educational Levels^a^ for Asthma and Atopy Combined Phenotype, Before and After Simultaneous Adjustment for Groups of Exposures (*n* = 6,378^b^), Avon Longitudinal Study of Parents and Children, 1991–1999

Model	Asthma Alone		Asthma and Atopy		Atopy Alone	
	aMOR^c^	95% CI	aMOR^c^	95% CI	aMOR^c^	95% CI
Adjusted for age and sex	1.50	1.21, 1.87	1.06	0.82, 1.36	0.80	0.66, 0.98
Additional adjustments						
Life-course exposure to tobacco smoke^d^	1.28	1.02, 1.60	1.05	0.81, 1.38	0.85	0.69, 1.05
Life-course exposure to tobacco smoke^d^ and maternal age at delivery	1.16	0.91, 1.47	1.01	0.76, 1.33	0.87	0.70, 1.08
Life-course exposure to tobacco smoke^d^ and breastfeeding	1.18	0.94, 1.48	1.07	0.81, 1.40	0.87	0.70, 1.08
Life-course exposure to hygiene hypothesis exposures^e^	1.36	1.08, 1.71	1.15	0.88, 1.51	0.92	0.75, 1.14
Life-course exposure to hygiene hypothesis exposures^e^ and maternal age at delivery	1.20	0.95, 1.52	1.11	0.84, 1.47	0.96	0.77, 1.20
Life-course exposure to hygiene hypothesis exposures^e^ and breastfeeding	1.23	0.97, 1.55	1.18	0.89, 1.55	0.96	0.77, 1.19

Abbreviations: aMOR, adjusted multinomial odds ratio; CI, confidence interval.

^a^ High indicates a university degree; low indicates O levels or below (education attained at 16 years of age, including vocational education).

^b^ Generated from multiple imputation with 25 imputed data sets.

^c^ Compared with group that had no asthma and no atopy.

^d^ Exposure to tobacco smoke includes maternal smoking, paternal smoking, and exposure to environmental tobacco smoke in pregnancy and childhood.

^e^ Hygiene hypothesis exposures included the presence of older children at home at the time of birth, a crowding index (the number of people in the household divided by the number of rooms), and the presence of a pet during pregnancy and childhood.

#### Wheezing and other phenotypes

Persistent wheeze—wheezing that starts early in life and continues throughout childhood—was more common among children with a poor socioeconomic background. Maternal smoking during pregnancy, crowding during pregnancy or childhood, cotinine levels, exposure to bleach or dye products in pregnancy, and breastfeeding duration were the main exposures that individually (29) diminished the association towards the null. Adjustment for all life-course exposure to tobacco smoke resulted in the association crossing the null value of no association (for low vs. high SEP, odds ratio = 1.15, 95% CI: 0.75, 1.77; 67% attenuation; Web Table 3), but this was entirely due to cotinine levels measured in childhood (for all life-course exposure to tobacco smoke without including cotinine levels in the model, odds ratio = 1.51, 95% CI: 1.03, 2.20). Adjustment for all life-course hygiene-related variables also partly attenuated the association (for low vs. high SEP, odds ratio =1.37, 95% CI: 0.99, 1.90; 19% attenuation). There was no specific pattern of groups of exposures (smoking or hygiene hypothesis) attenuating the inequality observed in phenotypes based on reported symptoms (asthma and eczema in the past 12 months) (Web Table 3).

#### Lung function

Socioeconomic patterning of lung function measures was observed for FEV_1_ but not forced vital capacity or midbreath forced expiratory flow (Table 3). The differences in FEV_1_ by paternal educational level were small. There was no clear pattern of variables attenuating this association, and inclusion of most variables resulted in the association crossing the null (data not shown).

## DISCUSSION

In this contemporary birth cohort, we have demonstrated that some but not all asthma phenotypes are unequally distributed across socioeconomic groups. This has highlighted the clinical and biological attributes that, by the age of 8 years, are susceptible to socioeconomic influences. Failure to stratify asthma based on these characteristics could contribute to inconsistent reports of inequalities. The combined asthma-atopy phenotype captured the greatest variation. Although asthma is often described as an allergic disease, particularly among children, a large proportion of asthma is not associated with atopy (30, 31) or might not be related to allergic inflammation processes in the airway (7). Specific exposures were related to the socioeconomic patterns found in this phenotype. Other disease phenotypes based on wheezing patterns, lung involvement, or bronchial reactivity were not socioeconomically patterned or had only small differences across socioeconomic groups. Future work should ascertain whether other clinical or biological characteristics become socioeconomically patterned in adolescence and adulthood, because such factors might be key to understanding and tackling future life-course inequalities of these conditions.

### Socioeconomic patterning of different disease phenotypes

Disease phenotypes based on the combination of doctor's diagnosis of asthma and a finding of atopy were associated with the greatest and opposing socioeconomic patterns across categories. Asthma alone was more likely among children from poorer socioeconomic backgrounds, and atopy alone was more likely among better-off children. We used an objective test of atopy (positive skin-prick test result) to avoid a potentially biased self-report of allergic conditions. A higher prevalence of allergies and atopy among people with higher SEPs has been reported in some but not all studies (18, 31). A similar overall allergen exposure can hide the heterogeneity in the sensitization to specific allergens in children or adults, which may vary in different countries. In the United States, the majority of children from poor socioeconomic backgrounds were sensitized to cockroach allergens, but fewer were sensitized to dust mite allergens (32). In our sample, sensitization to the German cockroach was uncommon (only 3 children). The role of this heterogeneity in modulating the risk of developing asthma is not known, but it is plausible that different types of allergen sensitization target different organs and result in different clinical symptoms.

Wheezing phenotypes, which are based on the onset of and time variation in wheezing symptoms, were important in the characterization of the natural history and clinical evolution of symptoms but did not vary across socioeconomic groups, with the exception of persistent wheeze. Persistent wheeze was more frequent among children from a poor socioeconomic background. Although we cannot rule out overreporting of wheezing symptoms among low-SEP groups, this likely identifies a more severe form of disease, which has consistently been associated with low SEP in the literature (16, 33).

Finally, objective tests of lung involvement (spirometry and bronchial reactivity tests) did not indicate differences across socioeconomic groups with the exception of a slightly lower FEV_1_ among children whose fathers had a lower educational level. This suggests that the SEP patterning in symptoms and doctor diagnosis of asthma have not yet translated into socioeconomic differences in lung function or bronchial hyperresponsiveness in this cohort of young children. Transition to adolescence and adulthood could bring new exposures or changes in the clinical and biological characteristics that are susceptible to socioeconomic variation. Future research should be conducted to ascertain whether this is the case to understand how inequalities in these conditions change over the life course.

### Exposures attenuating the socioeconomic patterning

No single exposure importantly attenuated the SEP patterns. Furthermore, a different set of exposures attenuated the opposing socioeconomic patterns observed in the asthma and atopy phenotypes.

#### Exposure to tobacco smoke

Our study identified exposure to tobacco smoke as the main avoidable exposure related to the asthma alone phenotype. Its risk appeared to start in utero and continue throughout childhood. The risk was related to all sources of exposure: maternal, paternal, and environmental. There is convincing evidence that exposure to tobacco smoke during pregnancy plays a role in airway development (2, 34) and in the development of wheezing and asthma in the offspring of smokers (35–39). Exposures to parental smoking and environmental tobacco smoke in childhood are associated with asthma and asthma symptoms (39, 40) and with reduced lung function in early adulthood (41). Quasi-experimental data from smoking bans have shown a subsequent decrease in asthma hospitalizations in children (42) and adults (43).

In the present study, exposure throughout the life course, rather than at specific time periods, contributed to inequalities and to the overall burden of this asthma phenotype. Our results highlight the importance of preventing smoking during pregnancy, which was the time of exposure that seems to account for a greater proportion of inequalities in the asthma alone phenotype. This is likely because in our population, the socioeconomic differences between those who smoked during pregnancy and those who did not were greater than the differences between those who were exposed to smoking during childhood and those who were not.

Finally, exposure to tobacco smoke was associated with lower odds of atopy alone, although it had a negligible role attenuating the lower risk of atopy alone among children of low SEP. Our results contrast with a small but higher risk of self-reported allergic conditions associated with active and passive exposure to tobacco smoke reported in a recent meta-analysis (44).

Future work using Mendelian randomization of smoking-related variants and cord blood cotinine levels could help to establish the importance of in-utero exposure to tobacco smoke in the asthma alone phenotype and could help establish a potentially causal association with an atopy phenotype (45).

#### Exposures related to the hygiene hypothesis

Factors traditionally related to the hygiene hypothesis (number of older children in the household, crowding, and owning a pet) tended to account for the higher odds of atopy alone among children of higher SEP. Whether the hygiene hypothesis first proposed by Strachan (46), which suggests that greater microbial exposure in early life reduces the risk of atopy, is the mechanism responsible for a higher risk of allergy among people with high SEP remains inconclusive (47). We found this to be the case in a historical cohort of university students in whom self-reported asthma and atopy occurred before the well-reported rise in asthma and atopy in the United Kingdom (48, 49). This result suggests that mechanisms driving the hygiene hypothesis must already have existed and persist today, despite important changes in socioeconomic and hygienic conditions in the United Kingdom. Recently proposed mechanisms include biome depletion due to hygiene advances achieved during the 20th century (50).

Hygiene hypothesis exposures did partly attenuate the socioeconomic patterning of asthma alone, although not to the null. The attenuation was mainly shown with the crowding variable, which was correlated with higher exposure to tobacco smoke.

#### Maternal age and breastfeeding

Older maternal and paternal ages were associated with lower odds of asthma alone and higher odds of atopy alone. These associations were not attenuated by birth order. Maternal age has been previously related to wheezing and asthma, although there is no strong evidence or rationale for a causal association (51, 52).

The evidence of a protective association of breastfeeding with asthma and atopy is conflicting and remains inconclusive (53–57). Women with high socioeconomic status in high-income countries are more likely to breastfeed than are women with low socioeconomic status; study designs that allowed better control of this selection bias, such as a sibling comparison (58) and a cluster randomized trial of a breastfeeding intervention (59), did not provide evidence of a protective association of breastfeeding with asthma (59, 60) or sensitization (59).

There is no clear biological mechanism that explains these associations, and older maternal and paternal ages, as well as breastfeeding, are very strongly correlated with higher SEP. There is also a simultaneous protective and risk-factor “role” associated with these factors depending on disease phenotype. We hypothesize that the associations are likely to be due to residual confounding.

### Strengths and limitations

We investigated inequalities in disease risk in children with various asthma and atopy phenotypes to identify a way to reduce these inequalities but also to understand the mechanisms of disease etiology. To this end, we evaluated an extensive list of asthma phenotypes being used in clinical and research settings. Other strengths include the lengthy list of SEP measures and the large number of potentially mediating exposures ascertained prospectively. The main limitation of our study is the loss of participation during follow-up. To account for this, we used missing data techniques.

### Conclusions

Including markers of atopy is key to understanding the socioeconomic patterning of asthma phenotypes. The inequality in the rates of asthma alone among poor children and the overall burden of this disease phenotype can be prevented by eliminating exposure to tobacco smoke throughout the life course, including in utero. The inequality in rates of atopy alone among well-off children remains complex, with hygiene hypothesis variables as the main mediators. Understanding the life-course development of atopy—and whether specific versus generic sensitization results in different atopic symptoms and asthma risk over the life course—is likely to shed light into its disease mechanism and mediating exposures.

## Supplementary Material

Web Material
