# Supplementary material for: Childhood Wheezing, Asthma, Allergy, Atopy, and Lung Function: Different Socioeconomic Patterns for Different Phenotypes
Source: Am J Epidemiol. 2015 Oct 6;182(9):763–74. doi: 10.1093/aje/kwv045 (PMC4617295; doi:10.1093/aje/kwv045)
Supplement: Web Material [file supp_182_9_763__index.html]

Childhood Wheezing, Asthma, Allergy, Atopy, and Lung Function: Different Socioeconomic Patterns for Different Phenotypes — Childhood Wheezing, Asthma, Allergy, Atopy, and Lung Function: Different Socioeconomic Patterns for Different Phenotypes — Web Material 

# Childhood Wheezing, Asthma, Allergy, Atopy, and Lung Function: Different Socioeconomic Patterns for Different Phenotypes

## Web Material

Web Material

- Web Material - Pdf file
