# Supplementary material for: Childhood Wheezing, Asthma, Allergy, Atopy, and Lung Function: Different Socioeconomic Patterns for Different Phenotypes
Source: Am J Epidemiol. 2015 Oct 6;182(9):763–74. doi: 10.1093/aje/kwv045 (PMC4617295; doi:10.1093/aje/kwv045)
Supplement: Web Material [file supp_kwv045_kwv045supp.pdf]

Web Appendix 1.

Description and analytical categories of life course parental and child factors and exposures.

Maternal and paternal age (years) at delivery were calculated using mother's date of birth (provided at enrolment) and paternal date of birth (reported at 8 weeks of the pregnancy) and the child's date of birth. *Gestational age* (weeks) was obtained from obstetric records. *Birth weight* was obtained from obstetric records. Birth weight in grams was standardised by gestational age (in weeks) and gender to create birth weight z-scores. The proportion of *children born in a household with no other children* at birth was measured at 6 months of age based on the response to the question of "How many other children live with you?" (reference group: children living with 1 or more other children in the household). *Breastfeeding duration* was based on questionnaire data collected at 6 months and categorised as "never or less than 1 month", "greater than 1 month but less than 3 months" and "more than 3 months" (reference group).

Maternal smoking during pregnancy was categorized as the number of trimesters the mother smoked "never, "smoked 1 or 2 trimesters" and "smoking throughout pregnancy (3 trimesters)" using smoking information collected at 18 weeks (number of cigarettes smoked in the first trimester of pregnancy and during the last 2 weeks) and at 8 weeks after birth (about the number of cigarettes smoked during the last 2 month of pregnancy). *Maternal smoking during childhood* was categorized as smoker or non-smoker using the number of cigarettes the mother smoked at 85 months. *Paternal smoking during pregnancy* was categorized as "never" or "smoked 1 or 2 trimesters" using information reported by the child's mother at 18 weeks of pregnancy and at 8 weeks after birth (number of cigarettes partner smoked during the last 2 month of pregnancy). *Paternal smoking during childhood,*

reported by the mother, was categorized as smoker or non-smoker using the number of cigarettes smoked during week and weekend days at 73 months. *Environmental tobacco smoke (ETS)* exposure was ascertained in *gestation* (32 weeks) and *childhood* (77 months) based on the following question "how often during the day you are in a room or enclosed place where other people are smoking?" and categorised as "never or less than 1 hour" and "1 hour or greater". Finally, *cotinine levels* were measured from blood samples provided at age 7-8 years. Each sample was run in duplicate and the average of these two measures was taken. In order to determine cotinine levels the absorbance at a wavelength of 450nm was measured in ng/ml. The assay uses a cubic spline curve to extrapolate and calculate cotinine concentration from the absorbance.

Use of bleach or hair dye was measured at 8 weeks of the pregnancy and was categorized as "Daily or most days" or "About once a week or less" (reference group). A *crowding index* was calculated by dividing the number of people in the household by the number of rooms, both reported by questionnaire. These were measured at the beginning of the pregnancy (8 weeks) and during early childhood (21 months). An ordinal variable was derived with groupings of <0.5 (reference group), 0.5-<0.75, 0.75-<1 and 1 or more. *Day care attendance* was assessed at 15 months by questionnaire and categorized as "not being in childcare" (reference group), "childcare at someone else home" or "being at nursery". At 85 months the mother reported about the child's health. A positive response to *chest infections in the last 12 months* is used in these analyses.

Bedroom temperature in winter (8 weeks pregnancy) or coldest time of the year (85 months childhood) were categorised as "cold or very cold" or "very warm, warm or about right" (reference group). *Presence and level of mould* in the house was categorized as "fairly or very serious" or "none or not serious" (reference level) through questionnaires at 8 weeks gestation and 85 months in childhood. *Pet ownership* was calculated based on positive

responses to owning either cats, dogs, rabbits, rodents, birds or other pets at pregnancy (8 weeks) and childhood (85 months). *Exposure to pests in the home* (8 week pregnancy and 85 month childhood) was determined if the mother reported that the home was invaded or there was dirt in the garden/balcony/yard related to rats, mice, pigeons or cockroaches. *Gas cooking* was ascertained if the mother positive response to "Do you use gas for cooking (rings and/or oven)?" at 8 week pregnancy at 85 months childhood.

Finally, *maternal and paternal history of asthma, eczema and hay fever* were reported by questionnaire administered at 12 weeks gestation.

Statistical analysis

Multivariable multiple imputation

There was a varying degree of missing values due to the long term follow-up. In order to increase efficiency and minimise selection bias we used multivariable multiple imputation to impute missing variables for participants considered to be eligible. We used chained equations(1), where a separate regression model was specified for each missing variable which included all life course exposures and characteristics, outcomes and potential predictors of missing data(2). We generated 25 imputed datasets and combined the multiple results into one multiple-imputation inference using Rubin's combining rules (3). Supporting Table S1 provides the percentage imputed for each variable and the mean value/distribution for continuous/categorical variables in the imputed and the original dataset.

Web Table 1. Summary statistics of relevant variables in the imputed dataset compared to the observed dataset. ALSPAC, 1991-1999.

**Outcomes/exposures and other characteristics (time of measurement)**	**Imputed %**	**Distribution mean (SE) and % for categorical variables in:** Imputed dataset	Observed dataset
Sex, % boys	0	-	50.2
Age at clinic 7	0	-	7.5 (0.2)
**Outcomes**			
Asthma in the last 12 months (91m)	12.8	11.2	11.1
Eczema in the last 12 months (91m)	12.8	17.6	17.7
Hay fever in the last 12 months (91m)	13.1	9.2	9.2
Ever Doctor diagnosis of asthma (91m)	13.3	19.8	19.6
Atopy (positive Skin Prick Test) (clinic at 7-8)	15.3	20.5	20.5
FVC z-score, (clinic at 7-8)	20.2	0.02 (0.01)	0.02 (1.0)
FEV1 z-score (clinic at 7-8)	21.4	0.03 (0.01)	0.03 (1.0)
FEF25-75 z-score (clinic at 7-8)	20.2	0.03 (0.01)	0.03 (1.0)
Bronchial hyper-responsiveness (clinic at 7-8)	14.9	16.8	16.7
**Exposures/Characteristics**			
Paternal education, % High (32 weeks pregnancy)	0	-	23.8
Maternal asthma (12 weeks pregnancy)	2.8	11.4	11.4
Paternal asthma (12 weeks pregnancy)	25.4	12.9	12.9
Maternal eczema/hay fever (12 weeks pregnancy)	4.1	45.8	45.9
Paternal eczema/hay fever (12 weeks pregnancy)	26.9	39.7	39.9
**During pregnancy**			
Maternal smoking – Throughout 3 trimesters	4.5	10.8	10.3
Paternal smoking –1or2 trimesters	6.3	30.5	30.5
Environmental tobacco smoking ≥1 hour/day (32 weeks)	16.8	42.8	44.2
Crowding ≥ 1 (8weeks)	2.5	12.3	12.3
Pet ownership, % yes (8weeks)	1.6	57.6	57.7
Pests exposure at home (8weeks)	1.6	14.3	14.3
Gas cooking	2.7	53.9	53.9
Bleach/dye, % ≥most days (8 weeks)	1.2	16.7	16.6
Temperature bedroom –% Cold or v. Cold (8 weeks)	3.1	15.7	15.6
Mould, %fairly or v. serious (8 weeks)	2.8	1.8	1.8
**At birth**			
Maternal age	0	-	29.3 (4.4)
Paternal age	29.2	31.6 (0.07)	31.6 (5.5)
Gestational age (weeks)	0	-	39.8 (1.3)
Birth weight z-score	1.1	0.07 (0.01)	0.07 (0.98)
**Post-natal**			
Other children household when born, % none	4.7	44.9	44.8
Breastfeeding, % never or <1month (6 months)	6.1	32.9	32.4
Maternal smoking (85months)	12.4	17.2	16.2
Paternal smoking (73months)	19.9	23.1	21.1
Environmental tobacco smoking ≥1hour/day (77months)	13.8	14.5	13.7
Cotinine level	32.3	1.2 (0.02)	1.2 (1.2)
Day care attendance, % nursery (15 months)	6.1	6.9	7.0
Crowding ≥ 1 (21 months)	11.5	19.6	19.3
Pet ownership (85 months)	13.2	72.2	71.8

**Outcomes/exposures and other characteristics (time of measurement)**	**Imputed %**	**Distribution mean (SE) and % for categorical variables in:**	
		Imputed dataset	Observed dataset
Pests exposure at home (85 months)	4.6	15.4	15.3
Gas cooking	11.5	61.7	61.9
Temperature child bedroom –% Cold&v. Cold (8 weeks)	12.1	5.4	5.3
Mould in bedroom, %serious or v. serious (85 months)	55.2	2.9	2.8
Chest infection last 12 months (85 months)	12.2	10.5	10.3

Web Table 2. The association (age and sex adjusted) of life course exposures and characteristics with the combined asthma-atopy phenotypes (imputed dataset n=6378[1]). ALSPAC, 1991-1999.

	Multinomial odds ratios and 95% confidence intervals[2]:			
	Asthma alone	Asthma & atopy	Atopy alone	Heterogeneity[3]
**During pregnancy**				
Maternal age	0.95 (0.93, 0.97)	0.98 (0.96, 1.01)	1.02 (1.00, 1.03)	<0.0001
Paternal age	0.98 (0.96, 0.99)	0.99 (0.97, 1.01)	1.01 (1.00, 1.03)	0.002
Maternal smoking (ref -none)				
1 or 2 trimesters	1.19 (0.86, 1.65)	1.11 (0.75, 1.64)	0.69 (0.46, 1.03)	0.07
Throughout 3 trimesters	1.84 (1.45, 2.33)	1.05 (0.75, 1.47)	0.70 (0.52, 0.94)	<0.0001
Paternal smoking (ref -none)				
1-2 trimesters	1.34 (1.12, 1.60)	1.15 (0.92, 1.44)	0.98 (0.83, 1.17)	0.03
ETS (ref - ≤1 hour per day)				
>1 hour per day	1.62 (1.36, 1.94)	1.08 (0.87, 1.35)	0.85 (0.71, 1.01)	<0.0001
Crowding (Ref <0.5)				
0.50 to 0.75	1.06 (0.86, 1.29)	1.07 (0.84, 1.37)	0.90 (0.74, 1.09)	0.35
>0.75 to 1	1.13 (0.86, 1.48)	1.15 (0.84, 1.56)	0.82 (0.64, 1.07)	0.12
>1	1.55 (1.19, 2.03)	0.90 (0.62, 1.30)	0.69 (0.51, 0.93)	0.0001
Pests exposure – Yes vs No	1.10 (0.87, 1.37)	0.79 (0.57, 1.08)	0.95 (0.74, 1.21)	0.21
Pet ownership - Yes vs No	1.20 (1.01, 1.44)	0.79 (0.65, 0.97)	0.72 (0.61, 0.85)	<0.0001
Gas cooking - Yes vs No	0.95 (0.79, 1.13)	0.91 (0.74, 1.12)	0.98 (0.83, 1.16)	0.84
Bleach/dye (ref: ≤once /week)				
≥most days	1.54 (1.23, 1.93)	1.17 (0.89, 1.52)	0.87 (0.68, 1.12)	0.001
Bedroom temperature (Ref – Warm, v warm/ about right)				
Cold/ very cold	1.12 (0.88, 1.41)	1.06 (0.81, 1.39)	0.96 (0.76, 1.21)	0.59
Mould home (ref No/not serious)				
Fairly/v serious	2.31 (1.39, 3.85)	1.36 (0.66, 2.80)	0.57 (0.26, 1.26)	0.01
**Peri-natal**				
Gestational age (weeks)	0.92 (0.87, 0.99)	1.04 (0.96, 1.13)	1.08 (1.01, 1.16)	0.001
Birth weight (z scores)	0.89 (0.82, 0.97)	1.00 (0.91, 1.12)	1.00 (0.92, 1.09)	0.09
**Post-natal**				
No older children household	0.95 (0.80, 1.12)	1.17 (0.96, 1.43)	1.22 (1.04, 1.44)	0.05
Breastfeeding duration (ref >3months)				
1 to 3 months	1.28 (1.00, 1.63)	1.03 (0.77, 1.37)	0.83 (0.65, 1.06)	0.03
< 1 month	1.52 (1.26, 1.84)	0.95 (0.76, 1.20)	0.84 (0.70, 1.02)	<0.0001
Maternal smoking (ref– no)	1.49 (1.20, 1.85)	1.11 (0.85, 1.45)	0.92 (0.73, 1.15)	0.004
Paternal smoking (ref -no)	1.47 (1.21, 1.77)	1.15 (0.89, 1.49)	0.90 (0.72, 1.11)	0.001
ETS (ref - ≤1 hour per day)				
>1 hour per day	1.33 (1.06, 1.68)	0.71 (0.51, 1.00)	0.73 (0.54, 0.97)	<0.001
Cotinine level (ng/ml)	1.15 (1.08, 1.23)	0.96 (0.86, 1.07)	0.90 (0.82, 0.98)	<0.0001
Crowding (ref <0.5)				
0.50 to 0.75	1.10 (0.79, 1.51)	0.76 (0.54, 1.07)	0.70 (0.54, 0.92)	0.07
>0.75 to 1	1.09 (0.77, 1.54)	0.74 (0.51, 1.06)	0.62 (0.46, 0.84)	0.03
>1	1.54 (1.10, 2.17)	0.69 (0.47, 1.01)	0.57 (0.41, 0.78)	<0.0001
Gas cooking - Yes vs No	1.02 (0.85, 1.23)	1.04 (0.84, 1.29)	0.94 (0.79, 1.12)	0.69
Pet ownership - Yes vs No	1.06 (0.87, 1.30)	0.65 (0.53, 0.81)	0.66 (0.55, 0.79)	<0.0001
Pests exposure - Yes vs No	1.14 (0.90,1.45)	0.77 (0.57, 1.04)	0.94 (0.75, 1.18)	0.1

	Multinomial odds ratios and 95% confidence intervals[2]:			
	Asthma alone	Asthma & atopy	Atopy alone	Heterogeneity[3]
Day care attendance (ref No)				
Someone else home	0.83 (0.64, 1.08)	1.03 (0.78, 1.37)	1.00 (0.79, 1.26)	0.40
Nursery	0.81 (0.57, 1.15)	0.89 (0.59, 1.35)	0.92 (0.66, 1.28)	0.83
Chest infections - Yes vs No	5.50 (4.3, 7.03)	9.58 (7.35, 12.5)	1.69 (1.22, 2.35)	<0.0001
Bedroom temperature (Ref – Warm, v warm/ about right)				
Cold/ very cold	1.13 (0.78, 1.64)	0.75 (0.44, 1.28)	1.22 (0.86, 1.72)	0.26
Mould home (ref No/not serious)				
Fairly/v serious	1.60 (0.87, 2.92)	1.12 (0.52, 2.38)	1.14 (0.57, 2.29)	0.60
**Family history**				
Maternal asthma	1.70 (1.33, 2.17)	3.11 (2.43, 3.98)	1.26 (0.97, 1.64)	<0.0001
Paternal asthma	1.60 (1.22, 2.11)	2.48 (1.88, 3.28)	1.43 (1.05, 1.93)	0.01
Maternal eczema/hay fever	1.49 (1.25, 1.77)	2.06 (1.67, 2.54)	1.46 (1.24, 1.73)	0.02
Paternal eczema/ hay fever	1.10 (0.90, 1.35)	2.01 (1.61, 2.51)	1.44 (1.19, 1.74)	<0.001

[1] Generated from multiple imputation with 25 imputed datasets

[2]Compared with "No asthma and no atopy" group.

[3]Tests equality of coefficients across outcome groups

Web Table 3. Adjusted association of low paternal education (compared to high education) for asthma and eczema in the last 12 months, and persistent wheezing, before and after simultaneously adjusting for groups of exposures. ALSPAC, 1991-1999.

Model	Asthma last 12 months<br>OR[1] (95% CI)<br>n=6378	Eczema last 12 months<br>OR[1] (95% CI)<br>n=6378	Persistent wheezing*<br>MOR[1] (95% CI)<br>n=6253
**Age and sex adjusted**	1.32 (1.06, 1.65)	0.81 (0.68, 0.96)	1.46 (1.12, 1.92)
Additionally adjusted for:			
**Life course exposure to tobacco smoke** (maternal smoking, paternal smoking and ETS[1] in pregnancy and childhood)	1.25 (1.00, 1.56)	0.81 (0.68, 0.97)	1.15 (0.75, 1.77)
Life course exposure to tobacco smoke (as above) **+ maternal age at delivery**	1.24 (0.99, 1.56)	0.82 (0.68, 0.98)	1.16 (0.75, 1.80)
Life course exposure to tobacco smoke (as above) **+ breastfeeding**	1.21 (0.96, 1.52)	0.86 (0.72, 1.02)	1.04 (0.66, 1.64)
**Life course exposure to hygiene-hypothesis exposures** (older children at birth and crowding and owning a pet in pregnancy and childhood)	1.37 (1.09, 1.71)	0.84 (0.70, 1.00)	1.37 (0.99, 1.90)
Life course exposure to hygiene-hypothesis exposures **+ maternal age at delivery**	1.34 (1.06, 1.68)	0.84 (0.70, 1.00)	1.29 (0.92, 1.81)
Life course exposure to hygiene-hypothesis exposures **+ breastfeeding**	1.32 (1.05, 1.65)	0.89 (0.74, 1.06)	1.33 (0.95, 1.87)

* "Persistent wheezing" of the 6 wheezing phenotypes, compared with "Never/infrequent" wheezing group and using a child's phenotype probability as weights

[1] OR: Odds Ratio; CI: confidence Interval; MOR: Multinomial Odds Ratio; ETS: Environmental Tobacco Smoke

REFERENCES

1. Van Buuren S, Brand JP, Groothuis-Oudshoorn C, et al. Fully conditional specification in multivariate imputation. *Journal of statistical computation and simulation* 2006;76(12):1049-64.
2. Royston P. Multiple Imputation of Missing Values. *Stata Journal* 2004;4:227-41.
3. Rubin DB. *Multiple imputation for non-response in surveys*. New York: John Wiley & Sons; 1987.
